# Editorial: Sensory Ecology of Phenotypic Plasticity: From Receptors via Modulators to Effectors

**DOI:** 10.3389/fnint.2022.930390

**Published:** 2022-05-26

**Authors:** Linda C. Weiss, Anke Schwarzenberger, Sebastian Kruppert

**Affiliations:** ^1^Department of Animal Ecology, Evolution and Biodiversity, Ruhr-University Bochum, Bochum, Germany; ^2^Limnological Institute, University of Konstanz, Konstanz, Germany; ^3^Friday-Harbor-Laboratories, University of Washington, Seattle, WA, United States; ^4^Plant Biomechanics Group, Botanical Garden, University of Freiburg, Freiburg, Germany

**Keywords:** phenotypic plasticity, neuronal pathway, chemical cue, behavior, anthropogenic impact

Organisms live within spatially and temporally dynamic environments. In order to cope with such ever-changing conditions, phenotypic plasticity is a wide-spread mean that allows organisms with a given genotype to express environmentally adapted phenotypes (Figure 1). Adaptive phenotypes eventually increase organismal fitness levels and can therefore ultimately affect community structures.

**Figure 1:**
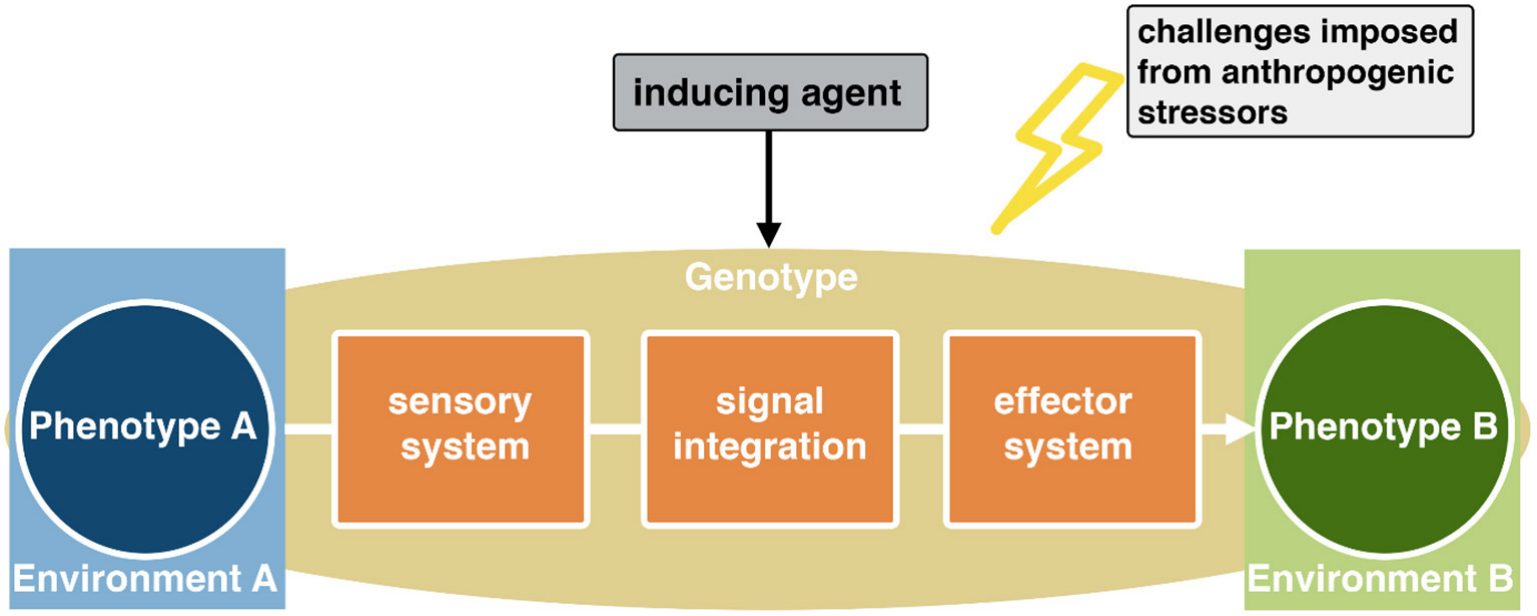
Phenotypic plasticity is realized through a complex cascade initiated by some kind of environmental challenge, which is indicated by a reliable cue. This cue has to be perceived by the organism through a distinct sensory system, and the signal has to be neuronally integrated to change developmental trajectories of effector sites.

In order to react plastically to changing environmental conditions organisms must be able to interpret the change in the environment and then express an alternative phenotype that increases organism fitness. Either individuals express a new phenotype themselves or produce a phenotypically adapted offspring generation.

Therefore, one central scope of recent scientific endeavors is to unravel, what kind of environmental information organisms perceive, and then how this information is perceived and integrated. In order to elucidate the adaptive value of the phenotypic response, comprehensive analyses of the phenotype (i.e., changes in behavior, morphology and life-history) and the molecular mechanisms underlying the phenotype (i.e,. short-term changes in gene expression, and long-term neo-/sub-functionalization of genes) are crucial.

In this collection, the contributions illuminate aspects of the biological pathways underlying the expression of adaptive phenotypes, spanning from the identification of critical environmental cues, the involved receptors to the interpreting nervous system. We welcomed articles covering aspects of neuronal transmission, neuronal plasticity and how developmental trajectories or gene functions are changed in order to express adaptive phenotypes. In order to comprehensively understand how fitness is optimized to changing environments we also called for articles that analyzed behavioral, morphological and life-history adaptations and how those increased organismal fitness.

Select predatory species can dine on specific, chemically defended prey species (Bernays et al., 2002; Hwang et al., 2007; Wink, 2018). Such biotic interactions play seminal roles, shaping community structure and function or triggering arms races and co-evolution especially among plants and herbivores (Anderson and Mitchell-Olds, 2011; Bruce, 2015). Zimmer et al. here present evidence that unlike chemical defense compounds, molecules uniquely associated with armored defenses can impact ecological relationships without the need for toxin resistance in source and/or consumer species. Liberated from these evolutionary constraints, chemosensory exploitation of prey body armor is likely widespread, acting as a critical ecological determinant.

Chemical detection goes both ways however, and prey species have been shown to react upon perceived predator presence; Daphniidae being a thoroughly studied example (for a review see Weiss and Tollrian, 2018). In fact, some kairomones (chemical cues released by predators) have been identified already (Weiss and Tollrian, 2018; Hahn et al., 2019). Hahn and von Elert show that a kairomone released by fish induces morphological changes in *Daphnia lumholtzi*. Given that this kairomone induces diel vertical migration in *Daphnia magna* (Hahn et al., 2019), these new findings present evidence that predator cues not only induce defenses in different species but also different types of defensive responses.

Furthermore, different environmental challenges are known to induce similar morphotypes in *Daphnia*. The most popular example being *D. cucullata* which reacts upon predation with a similar morphotype as it does for water turbulences (Laforsch and Tollrian, 2004a,b). Horstmann et al. here present a detailed analysis of these similar *D. cucullata* morphotypes suggesting that they are different levels of expression of the same “multitool”-morphotype that thwarts ingestion by predators and significantly reduces drag force allowing to minimize water disturbances that predators use to detect their prey. In addition to these multilevel defensive effects, the reduced drag force potentially increases *D. cucullata's* maneuverability adding to the advantages of this morphotype. In light of the previous and current investigations it appears likely that not a single positive effect, but an accumulation of favorable effects causes the distinct changes in morphology and swimming velocity.

Phenotypic plasticity is not limited to morphology alterations however, but also includes behavioral alteration as reactions to changing environments. Álvarez and Koene are reviewing the role of cognition and decision making on mating choices and through that to species evolution. Focusing on studies from the field of sexual conflict they show that cognition is an often time overlooked factor in analyses of mating behavior.

Besides natural environmental cues, anthropogenic stressors directly affect organism or influence species interactions. Anthropogenic stressors are very diverse and include Artificial Light At Night (ALAN; perceived by light receptors) and eutrophication with the occurrence of toxic cyanobacteria (detected by chemosensors). Cremer et al. combine both stressors and show that Daphnia are sensitive both toward dietary cyanobacterial chymotrypsin inhibitors and ALAN. The observed increase in chymotrypsin activity might be an artifact of ALAN-treatment. However, in environments with rising ALAN (that might support the growth of cyanobacteria) this increase of chymotrypsin activity (which had also led to enhanced growth) might be beneficial: If Daphnia are able to grow on toxin-producing cyanobacteria they might be able to outcompete other species that are not able to use this food-source.

Competition for resources is ubiquitous and has in some cases led to the evolution of resource polyphenism (RP), i.e., environmentally triggered alternative phenotypes showing differential use of niches or resources. For this developmental response different sensors are necessary in order to correctly assess the organisms' environment by making use of chemical and tactile information. Although not much empirical information is available on mechanisms used for this assessment, Levis and Ragsdale gives an actual overview over the three proximate mechanisms of RP and a summary of open questions addressing the molecular basis of developmental switching leading to RP.

A molecular analysis by Janeschik et al. reveals the Pax2 orthologs Pax2.1 and Pax2.2 to be involved in the development of lateral eyes and sensory hairs in spiders. While Pax6 is required in for eye development in most bilaterian lineages (Quiring et al., 1994), Pax2 is known to be involved in the development of sensory hairs in *Drosophila melanogaster* (Halder et al., 1995). Janeschik et al. here present a striking example of how gene duplication can lead to sub- and neo-functionalization of genes resulting in (sense) organ diversification.

## Author Contributions

LW initiated and served as head topic editor of this special issue. SK and AS served as invited topic editors. Editorial of the submitted manuscripts was equally distributed between LW, AS, and SK. All authors listed have made a substantial, direct, and intellectual contribution to the work and approved it for publication.

## Funding

This work was funded by the grant DFG WE6019/2-1.

## Conflict of Interest

The authors declare that the research was conducted in the absence of any commercial or financial relationships that could be construed as a potential conflict of interest.

## Publisher's Note

All claims expressed in this article are solely those of the authors and do not necessarily represent those of their affiliated organizations, or those of the publisher, the editors and the reviewers. Any product that may be evaluated in this article, or claim that may be made by its manufacturer, is not guaranteed or endorsed by the publisher.
